# TAp63: The fountain of youth

**DOI:** 10.18632/aging.100095

**Published:** 2009-10-21

**Authors:** Xiaohua Su, Elsa R. Flores

**Affiliations:** Department of Molecular and Cellular Oncology, Graduate School of Biomedical Sciences, The University of Texas M.D. Anderson Cancer Center, Houston, TX 77030, USA

**Keywords:** TAp63, aging, genomic instability, stem cells

## Abstract

The mechanisms controlling organismal aging have yet to be clearly defined.  In our recent paper [1], we revealed thatTAp63, the p53 family member, is a critical gene in preventing organismal aging by controlling the maintenance of dermal and epidermal precursor and stem cells critical for wound healing and hair growth.  In the absence of TAp63, dermal stem cells (skin-derived precursors or SKPs) in young mice are hyperproliferative.  As early as one month of age, SKPs and epidermal precursor cells exhibit signs of premature aging including a marked increase in senescence, DNA damage, and genomic instability resulting in an exhaustion of these cells and an overall acceleration in aging.  Here, we discuss our findings and its relevance to longevity, regenerative medicine, and tumorigenesis.

## *TAp63* maintains adult stem cells

**Figure 1.**
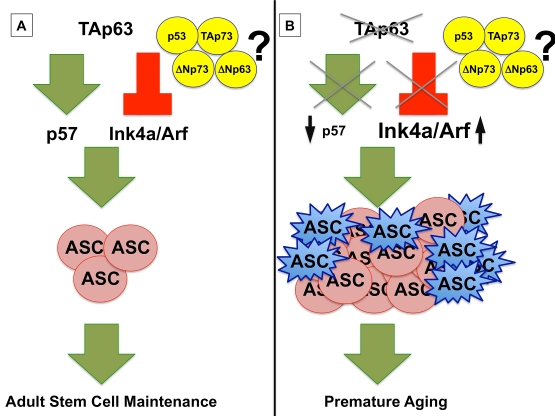
(**A**) TAp63 maintains adult stem cells (ASC) by transcriptionally activating *p57* and repressing *Ink4a/Arf,* preventing premature aging.  (**B**) In the absence of *TAp63, p57* mRNA levels are low, leading to hyperproliferation of ASCs (shown in pink), and *Ink4a/Arf* levels are high, resulting in a concomitant senescence of ASCs (shown in blue) and a premature aging phenotype in *TAp63* deficient mice.  The interplay of the *p53* family, including *TAp73, ΔNp73,* and *ΔNp63,* remains to be elucidated.

The mysterious mechanisms that regulate aging are an area of active research.  The induction of senescence or apoptosis in stem and progenitor cells is thought to trigger premature organismal aging [2].  Consistent with this idea, we found that the *TAp63-/-* mice had a significantly shortened life span compared to its wild-type littermates [1].  These mice exhibited phenotypes associated with premature aging including kyphosis, impaired wound healing, alopecia, epithelial and muscular atrophy, and chronic nephritis.  These phenotypes suggest a critical role for *TAp63* in the maintenance of adult stem cells in multiple epithelial and non-epithelial tissues.  Indeed, we found that *TAp63* maintains dermal stem cells by transcriptionally activating the cyclin dependent kinase inhibitor, *p57,* thereby preventing hyperproliferation of these cells (Figure 1A) [1,3].  Similar to the phenotype identified in dermal and epidermal progenitor and stem cells, other adult stem cells in the *TAp63-/-* mice may be hyperproliferative early in life and through similar senescence mechanisms that we delineated may result in a depletion of these stem cells and premature organismal aging (Figure 1B) [1].

## The complex roles of the *p53* family in aging

Increased p53 activity has been previously implicated in aging [4,5].  Although some mouse models with increased p53 activity exhibit signs of premature aging, others show conflicting results [6,7].  The important difference between these models is the alleles of *p53 *present in these mice.  The mice exhibiting signs of premature aging contain truncated p53 mutants [4,5] while those that display a normal lifespan upregulate p53 by other mechanisms, such as the expression of a *p53* transgene in addition to the endogenous *p53* alleles or a hypomorphic allele of *mdm2 *[6,7].  One potential explanation of the discrepancy in the phenotypes of these mice is that TAp63 interacts with point mutant p53 rendering TAp63 functionally inactive.  Consequently, mice expressing mutant p53 would exhibit phenotypes similar to those observed in the *TAp63-/-* mice.  Previous studies have shown this to occur in the context of tumorigenesis and metastasis [8,9].   Mice engineered to express point mutants of p53 in Li-Fraumeni Syndrome inactivate p63 and p73 in tumors by binding to them and preventing the transactivation of their target genes [8,9,10].  These mouse models exhibit a metastatic phenotype similar to that observed in *p53+/-;p63+/-* and *p53+/-;p73+/-* mice illustrating an intricate relationship between the *p53* family members [11,12].

Yet, another unexplored and possible explanation is that expression levels of the p53 family members change in mice that lack one or more of the family members, i.e. gene compensation. Such family member compensation has been observed in other families of genes including the *Rb* family [13,14,15].  In mouse models expressing abnormally high levels of p53, TAp63 levels may be dampened commensurate with an increase in p53 protein expression.  p53 protein levels are known to be high in mice expressing mutated versions of p53 [8,9,10].  Thus, loss of *TAp63* in these mouse models may again result in an acceleration of organismal aging.  Furthermore, other isoforms of *p63* and *p73* have been implicated in premature aging [16,17]. Therefore, careful characterization of the expression of the other p53 family members, including the individual isoforms of p63 and p73, is necessary in mouse models expressing altered levels of p53 in order to understand the complex interplay and potential compensation between the *p53* family members in processes that regulate longevity (Figure 1).

## Loss of *TAp63* triggers senescence and cannot be reversed by concomitant loss of *p53*

Interestingly and surprisingly, senescence triggered in *TAp63-/-* epidermal precursors is *p53*-independent. In fact, we found a higher proportion of senescent cells in *TAp63-/-;p53-/-* epidermal cells than in those lacking *TAp63* only, indicating that loss of *p53* does not bypass senescence in this tissue [1].  This further indicates that *TAp63* directly regulates senescence in epidermal precursor cells by transcriptionally repressing *Ink4a* and *Arf* as has been observed in the epidermis of mice deficient for *p63 *[1,18].    The mechanisms employed by *TAp63* to induce senescence have important implications for deciphering its role as a tumor suppressor gene.

## *TAp63* is induced in response to stress

*p63* evolved to have several isoforms that can be divided into two categories: the TA (transactivation competent isoforms) and the ΔN (those that lack the transactivation domain).  The most highly expressed isoforms of p63 in the skin are the ΔNp63 isoforms, thus the prevailing view is that ΔNp63, and more specifically ΔNp63α, are the isoforms that play critical roles in maintaining the epidermis [19,20]. However, it is important to note that the TAp63 isoforms structurally resemble p53 and have been shown in other systems to be induced in response to DNA damage and stress [21,22]. Importantly, although TAp63 protein expression is undetectable in the normal epidermis, we found that TAp63 expression increased dramatically in response to stress induced by wounding, indicating that much like *p53*, *TAp63* serves to protect cells from damage [1].  This is a novel and unrecognized role for *TAp63* in maintaining the dermis and the integrity of the epidermis.

## *TAp63:* The key to longevity?

Mice lacking *TAp63* also develop severe skin erosions that do not heal [1].  These erosions result from trauma or ruptured blisters that form in the majority of *TAp63-/-* mice.  The failure of these mice to appropriately heal their wounds results from a depletion of SKP cells known to be required for wound healing [1]. Additionally, the *TAp63-/- *mice exhibited patches where there was a diminution in the number of hair follicles resulting in alopecia in these mice.  Some of these defects are similar to those seen in patients with Hay-Wells syndrome or ankyloblepharon-ectodermal dysplasia-clefting (AEC) syndrome [23]. These pa-tients develop alopecia and skin erosions with impaired wound healing indicating that the *TAp63-/-* mouse may be useful as a preclinical model to test therapies for these disfiguring and painful diseases.

In addition, given the critical function of *TAp63* in wound healing and hair growth, reactivation of *TAp63 *in tissues of patients with degenerative diseases has important therapeutic implications not only in patients with AEC syndrome but also in those with impaired wound healing, like diabetes. Important areas for future investigation include developing models and therapies whereby *TAp63* can be reactivated in adult dermal stem cells to determine whether senescence and premature aging can be reversed in these cells to aid in the wound healing process and hair regeneration.

## The impact of the *TAp63-/- *aging phenotype on cancer

*p63* is an important suppressor of tumorigenesis and metastasis; however, at first glance, the role of *p63* in senescence and aging may seem at odds with its role as a tumor suppressor.  It is important to note that adult dermal stem cells are initially hyperproliferative prior to acquiring a senescent phenotype (Figure 1B).   By extension, in tumor formation, cancer stem or precursor cells that lose *TAp63* may likewise be hyper-proliferative.  With the high levels of DNA damage and genomic instability that are detected in dermal and epidermal stem cells lacking *TAp63 *[1]*, *these cancer stem cells will likely acquire new mutations that allow escape from senescence, an ideal formula for tumor formation.  In addition to further investigation on how *TAp63* affects cancer stem cells, the milieu in which cancer cells reside must also be closely examined in the *TAp63-/-* mouse model.  Cancer incidence increases with age, and it is possible that the prematurely aged environment of the *TAp63-/-* mouse provides an ideal environment for tumor formation and metastasis. Further investigation on the effects of premature aging in the *TAp63* deficient mouse model on tumor formation is critical to obtain an understanding of the roles of *TAp63* as a tumor suppressor gene.

In summary, we have revealed a critical role for *TAp63*in preventing premature aging and further complexity of the *p53* family, underscoring a need to understand the family as a whole and its roles in human diseases. A clear understanding of the intimate and complex relationship between the *p53* family of genes is essential to target this pathway in degenerative diseases and tumorigenesis.
